# Y14 governs p53 expression and modulates DNA damage sensitivity

**DOI:** 10.1038/srep45558

**Published:** 2017-03-31

**Authors:** Chia-Chen Lu, Chi-Chieh Lee, Ching-Tzu Tseng, Woan-Yuh Tarn

**Affiliations:** 1 Taiwan International Graduate Program in Molecular Medicine, National Yang-Ming University and Academia Sinica, Taipei, Taiwan; 2 Institute of Biomedical Sciences, Academia Sinica, Taipei, Taiwan

## Abstract

Y14 is a core component of the exon junction complex (EJC), while it also exerts cellular functions independent of the EJC. Depletion of Y14 causes G2/M arrest, DNA damage and apoptosis. Here we show that knockdown of Y14 induces the expression of an alternative spliced isoform of p53, namely p53β, in human cells. Y14, in the context of the EJC, inhibited aberrant exon inclusion during the splicing of p53 pre-mRNA, and thus prevent p53β expression. The anti-cancer agent camptothecin specifically suppressed p53β induction. Intriguingly, both depletion and overexpression of Y14 increased overall p53 protein levels, suggesting that Y14 governs the quality and quantity control of p53. Moreover, Y14 depletion unexpectedly reduced p21 protein levels, which in conjunction with aberrant p53 expression accordingly increased cell sensitivity to genotoxic agents. This study establishes a direct link between Y14 and p53 expression and suggests a function for Y14 in DNA damage signaling.

Y14 (RBM8A) forms a heterodimer with Magoh, as a part of the exon junction complex (EJC) core. The EJC is loaded onto the spliced mRNA and serves as a platform for association of peripheral factors that contribute to mRNA maturation, including mRNA export and surveillance and translation control. In addition, the individual EJC components have specialized functions in various cellular processes^1^. For example, Y14/Magoh specifically interacts with the methylosome and mRNA decapping complex and thus may regulate the maturation of small nuclear ribonucleoproteins and mRNA stability, respectively^2^,^3^. In addition, Y14 may selectively promote mRNA translation via binding to the mRNA cap^4^, but its targets remain to be identified.

Although the EJC essentially acts at the post-splicing steps of mRNA metabolism, its core factors probably assemble on the precursor mRNA prior to exon ligation. The spliceosomal factor CWC22 initiates EJC assembly during splicing^5^. Depletion of the individual EJC components indeed has a great impact on alternative splicing^6^,^7^. Y14 particularly modulates alternative splicing of apoptotic genes, such as those encoding Bcl-x, Bim, and Mcl1^6^. Depletion of Y14 leads to the generation of Bcl-x/Bim/Mcl1 isoforms with pro-apoptotic activity. A more recent study showed that Y14 is required for efficient splicing of short introns in transcripts encoding mitotic factors^7^. Association of the EJC with spliced exons may facilitate short intron definition and splicing. The EJC also influences other types of alternative splicing, but the underlying mechanism(s) remains to be investigated.

Mammalian Y14 is essential for cell-cycle progression and cell viability. Depletion of Y14 results in cell-cycle arrest essentially at G2/M phase, and depletion also increases the population of sub-G1 cells that are prone to apoptosis^8^,^9^. Y14 may act as a regulator of the cell cycle and apoptosis, at least in part by regulating alternative splicing of mitotic and apoptotic factors^6^,^7^. Moreover, Y14 and Magoh have been identified in the centrosome, and depletion of either protein increases a population of cells with multipolar or monopolar centrosomes^10^. Therefore, Y14/Magoh may play a role in mitotic spindle formation, G2/M checkpoint control, or DNA damage signaling. Gene knockout studies reveal that Rbm8a is critical for neural progenitor proliferation and neuronal differentiation in the developing mouse brain likely through its role in cell-cycle control and apoptosis^8^,^11^,^12^ (see Results and Discussion).

While studying how Y14 modulates the cell cycle and cell viability, we observed that depletion of Y14 induced an alternatively spliced isoform of p53 in cultured human cells (see below). p53 has a variety of isoforms generated by alternative splicing or differential use of the promoter or translation start site^13^. The p53 isoforms exhibit overlapping or distinct functions with canonical p53, and some of them may interfere with p53 signaling and contribute to tumor formation. Here, we explored the role of Y14 in regulating p53 expression and the effect of Y14 depletion on cell sensitization to genotoxic stress.

## Results

### Depletion of Y14 induces a p53 isoform

**Figure 1:**
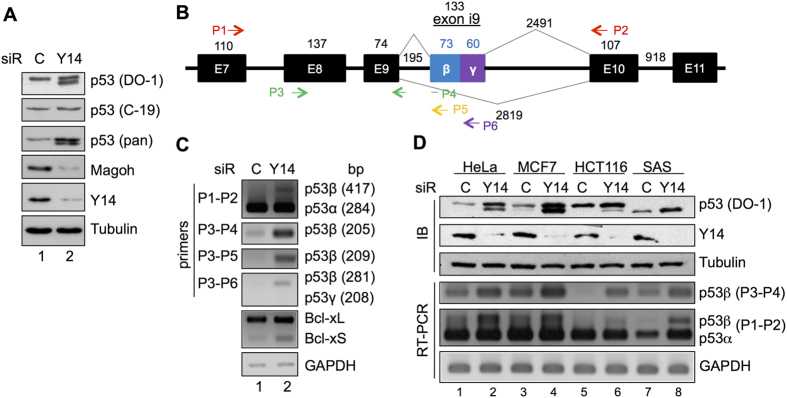
Depletion of Y14 induces the expression of a p53 isoform. (**A**) HeLa cells were transfected with control or Y14-targeting siRNA. Cell lysates were subjected to immunoblotting using antibodies against p53 (DO-1 for the N-terminal region, C-19 for the C-terminal region, and pan for entire p53), Magoh, Y14, and α-tubulin. (**B**) Schematic diagram of human TP53 and alternative splicing. Arrows indicate primers used for p53 detection in panel C. (**C**) RT-PCR of p53, Bcl-x, and GAPDH transcripts in control or Y14 siRNA-transfected HeLa cells. PCR product size is indicted in parentheses. (**D**) Immunoblotting (IB) of p53, Y14, and α-tubulin proteins and RT-PCR of p53 and GAPDH in control or Y14 siRNA-transfected cell lines as indicated.

To evaluate the cellular function of Y14, we depleted Y14 in HeLa cells by transient transfection of a short interfering RNA (siRNA) that targets Y14. The level of Y14 and its protein partner Magoh was significantly reduced by Y14 siRNA (Fig. 1A). As reported^9^,^10^, we observed that Y14 depletion caused cell-cycle arrest at G2/M phase and increased the sub-G1 population and apoptosis in HeLa cells (Supplementary Fig. 1). While examining the expression of cell-cycle and apoptotic factors, we fortuitously found that Y14 depletion induced a p53 band of smaller molecular size (Fig. 1A). This band was detected by a monoclonal antibody that recognizes an N-terminal peptide of p53 (DO-1), but not by a C-terminal peptide antibody (C-19), suggesting that the band represented a C-terminal deletion variant of p53 (Fig. 1A).

Using reverse transcription (RT)-PCR with different sets of primers (Fig. 1B), we confirmed that Y14 depletion induced the expression of the p53β isoform that includes exon i9, an aberrant exon residing in intron 9, and encodes a truncated protein with a C‑terminal sequence distinct from that of p53α (hereafter p53α refers to full-length p53) (Fig. 1C). No product corresponding to p53γ (208 bp) was detected (Fig. 1C, P3–P6). It has been reported that p53β can be induced by depletion of the splicing regulator SRSF3 or by treating cells with caffeine or digoxin^14^,^15^. This result suggested a role of Y14 in regulating p53 splicing. Moreover, Y14 depletion induced Bcl-xS, as previously reported^6^, confirming the effect of Y14 on splicing regulation. Notably, a mild increase of p53β mRNA did not reflect its protein level, which was almost comparable to that of p53α (Fig. 1A); this could be explained by the higher protein stability of p53β than p53α^16^.

**Figure 2:**
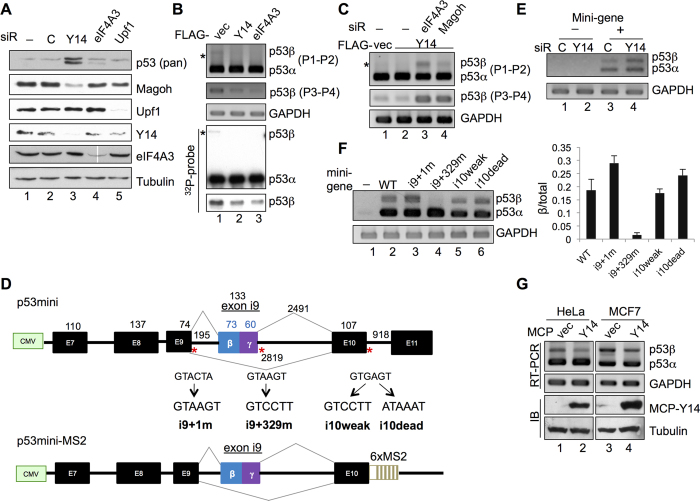
The EJC core prevents p53β expression. (**A**) Immunoblotting of p53, Magoh, Upf1, Y14, eIF4A3, and α-tubulin in mock-transfected or siRNA-transfected HeLa cells. (**B**) RT-PCR of p53 using primers P1–P2 and P3-P4 in HeLa cells transfected with the empty or FLAG-Y14 or eIF4A3 expression vector. GAPDH served as the control. Bottom panels show Southern blotting of the RT-PCR products of p53. Asterisks in both panels B and C indicate p53β. (**C**) HeLa cells were transfected with the empty or FLAG-Y14 expression vector without or with addition of siRNA targeting eIF4A3 or Magoh. RT-PCR of p53 and GAPDH was performed as in panel B. (**D**) Schematic diagrams for the wild-type and mutant p53 minigenes (p53mini) and the 6 × MS2-containing minigene (p53mini-MS2). Red asterisks indicate 5′ splice sites subjected to mutagenesis. (**E**) HeLa cells were transfected with control or Y14 siRNA with (lanes 3, 4) or without (lanes 1, 2) the p53 minigene. Primers used for RT-PCR of the p53mini transcripts were CMV and P2. (**F**) HeLa cells were mock transfected or transfected with a wild-type or mutant p53 minigene as indicated. RT-PCR was as in panel E. Bar graph shows p53β/total p53 ratio; average and standard deviation were obtained from three independent experiments. (**G**) HeLa or MCF7 cells were transfected with the p53mini-MS2 reporter and the MCP or MCP-Y14-HA expression vector. RT-PCR was as in panel E; MCP-Y14-HA was detected by immunoblotting (IB) with anti-HA.

Next, we depleted Y14 in a variety of cancer cell lines and examined p53 expression. Y14 depletion induced p53β protein significantly in breast cancer MCF7 cells and minimally in colon cancer HCT116 cells (Fig. 1D, lanes 4, 6). Because the TP53 allele in head and neck squamous carcinoma SAS cells contains a nonsense mutation in exon 10, Y14 depletion-induced p53β was indistinguishable from the truncated mutant p53 in SAS cells (lanes 7, 8). Nevertheless, we observed an increase in p53β mRNA expression, albeit to different extents, in all cell lines examined (Fig. 1D, RT-PCR). Additionally, we noted that the level of total p53 proteins increased after Y14 depletion, which was consistent among different cell lines (Fig. 1A,D). To examine how Y14 depletion causes p53 stabilization, we co-expressed FLAG-tagged p53 and HA-tagged ubiquitin in HeLa cells, and observed that anti-FLAG precipitated p53 was ubiquitinated after treatment of the cells with the proteasome inhibitor MG132 (Supplementary Fig. 2, lane 2). Ubiquitinated p53 was not detected in cells expressing the mutant ubiquitin (lanes 3, 4) or in Y14-depleted cells (lanes 5–8). Thus, Y14 depletion might prevent p53 ubiquitination. Moreover, the p53 protein level increase was not observed upon depletion of eIF4A3 (see below for Fig. 2), suggesting a specific role for Y14 in p53 protein biogenesis.

We noted that the mouse p53 gene has a shorter intron 9, which exhibits limited sequence homology to the 3′ part of its human counterpart (Supplementary Fig. 3A). A product equivalent to human p53β was not detected in Y14-depleted mouse neuroblastoma Neuro2a cells (Supplementary Fig. 3B). However, we noted that the level of p53 protein was raised in Y14-depleted Neuro2a cells, which was reminiscent of p53 activation in Rbm8a halopinsufficiency mouse embryos^12^, suggesting a conserved role for Y14 in p53 protein stabilization.

### The EJC core prevents p53β expression

Next, we examined whether depletion of any other EJC factor or disruption of nonsense-mediated mRNA decay (NMD) also induces p53β. The result showed that p53β was induced by deletion of eIF4A3 (Fig. 2A) or Magoh (Supplementary Fig. 4A) but not by depletion of the NMD factor Upf1 (Fig. 2A) or overexpression of the dominant-negative Upf1 (Supplementary Fig. 4B). Therefore, p53β was likely induced by depletion of the EJC but not by blockage of the NMD pathway. Although the level of p53β mRNA was extremely low, overexpression of Y14 or eIF4A3 reduced basal p53β expression (Fig. 2B). Knockdown of another EJC factor (eIF4A3 or Magoh) greatly reversed the effect of Y14 overexpression (Fig. 2C, lanes 3, 4), as expected. This result may moreover imply the concerted action of the EJC core factors in preventing exon i9 inclusion.

To further explore the mechanism of p53β expression, we inspected the sequence of intron 9 and observed that it has a weak authentic 5′ splice site (SS; +1 GTACTA) and a strong cryptic 5′ SS (*i.e*., downstream of exon i9; +329 GTAAGT) (Fig. 2D). Thus, we generated a minigene spanning exons 7 to 11 of the human TP53 gene to evaluate whether 5′ SS strength influences exon i9 inclusion (Fig. 2D, p53mini). As observed for endogenous p53, knockdown of Y14 induced exon i9 inclusion of the minigene reporter (Fig. 2E). We then created several 5′ SS mutants by site-directed mutagenesis. RT-PCR analysis revealed that a strengthened 5′ SS + 1 and a weakened 5′SS + 329 enhanced or abolished exon i9 inclusion, respectively (Fig. 2F, lanes 2–4).

We suspected that splicing of intron 10, which carries the strong 5′ and 3′ splice sites, may be faster than that of intron 9, and hence the EJC is recruited to prevent aberrant exon i9 inclusion. We therefore generated the mutant minigenes with a weakened or disrupted 5′ splice site of intron 10 (Fig. 2D, i10weak and i10dead). The result reproducibly showed that the i10dead mutant had increased exon i9 inclusion efficiency, although not in the i10weak mutant (Fig. 2F, lanes 5, 6). To further test our supposition that efficient intron 10 splicing suppresses exon i9 inclusion via recruiting an EJC, we generated a tethering reporter by replacing intron 10/exon 11 with a fragment containing six tandem repeats of the MS2 coat protein (MCP) binding sites (Fig. 2D, p53mini-MS2). The resulting reporter was co-transfected with the vector expressing an MCP-Y14 fusion into HeLa and MCF7 cells. RT-PCR analysis revealed that MCP-Y14 could reduce p53β expression from the minigene (Fig. 2G), indicating that Y14 association with a downstream exon—mimicking the anchor of an EJC—suppressed exon i9 inclusion. Hence, several lines of the data implied that EJC deposition modulates p53β expression (Fig. 2B,C,F,G).

### Camptothecin disrupts Y14 depletion-induced p53β expression

**Figure 3:**
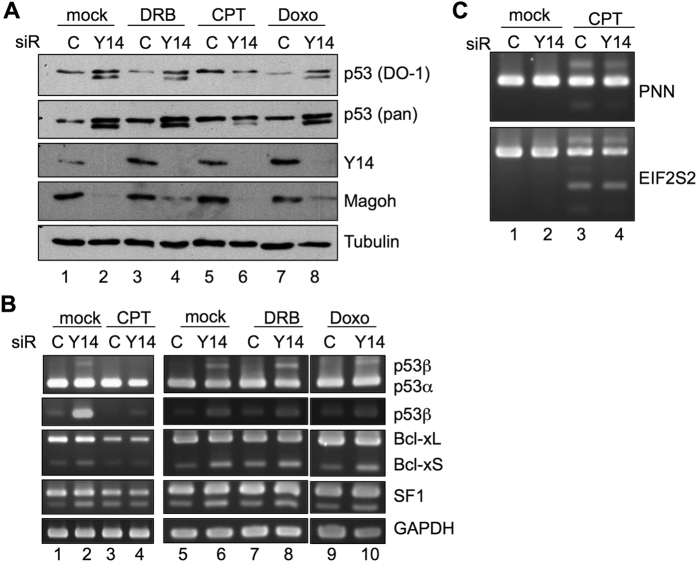
Camptothecin disrupts Y14 depletion-induced p53β expression. (**A**) HeLa cells were transfected with control or Y14 siRNA followed by treatment with compounds as indicated. Immunoblotting shows p53, Y14, Magoh, and α-tubulin. Doxo, doxorubicin. (**B**) RT-PCR of p53, Bcl-x, and SF1 isoforms in transfectants as in panel A. (**C**) RT-PCR of PNN and EIF2S2 isoforms in HeLa cells treated as in panel B.

Because the transcriptional elongation rate affects exon selection^17^, we tested whether the RNA polymerase II inhibitor 5,6-dichloro-1-β-d-ribofuranosyl-benzimidazole (DRB) influences p53β expression. We also examined two anti-cancer and DNA-damaging agents, camptothecin (CPT) and doxorubicin, that respectively inhibit DNA topoisomerase (TOP) I and II. CPT creates TOP1-DNA adducts, which also interfere with transcription elongation^18^. DRB and CPT have differential impact on splicing^19^,^20^,^21^. When Y14-depleted cells were treated with either of these compounds, immunoblotting revealed that CPT particularly reduced p53β protein (Fig. 3A). RT-PCR confirmed that CPT blocked the expression of the basal and Y14-induced p53β mRNA isoform (Fig. 3B, cp. lanes 1 and 3 in P3-P4). Moreover, CPT also eliminated the effect of Y14 depletion on two other targets, namely Bcl-x and SF1 (Fig. 3B, lanes 1–4). Nonetheless, DRB or doxorubicin had no such effects (Fig. 3B, lanes 5–10). Moreover, we examined two CPT target transcripts, EIF2S2 and PNN^20^. Y14 depletion neither affected their splicing (Fig. 3C, lane 2) nor altered the effect of CPT (lane 4). Thus, Y14 depletion and CPT treatment affected the splicing of overlapping, but still distinct, sets of transcripts. More importantly, our result indicated that CPT particularly reversed the effect of Y14 depletion in alternative splicing.

Notably, CPT preferentially affects the splicing of splicing factors, including Y14^20^ (Supplementary Fig. 5). CPT treatment likely yields a loss-of-function isoform of Y14 and thus impacts on the splicing of Y14 targets. However, this cannot explain how CPT specifically blocked Y14 depletion-induced alternative splicing. This intriguing result must be a topic of future investigation. Additionally, CPT exposure causes co-transcriptional R-loop formation^18^. The possibility of whether such a structure recruits a factor(s) to facilitate the selection of EJC-sensitive exons also remains to be tested by future experiments.

### The EJC ensures efficient translation of p53 mRNA

**Figure 4:**
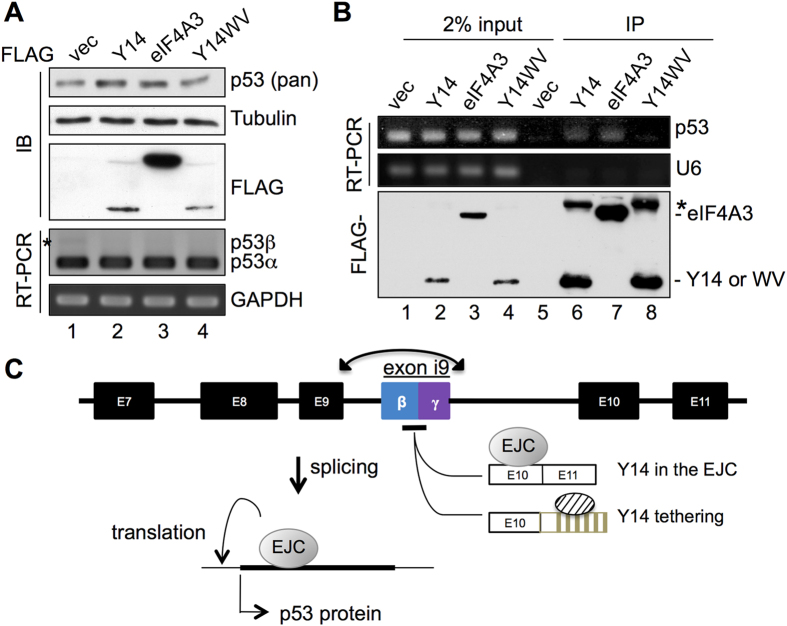
The EJC enhances p53 protein expression. (**A**) HeLa cells were transfected with the empty or FLAG-Y14 or eIF4A3 expression vector. Immunoblotting of p53, FLAG-tagged proteins, and α-tubulin, and RT-PCR of p53 and GAPDH are shown. Asterisk indicates p53β. (**B**) Immunoprecipitation was performed using anti-FLAG in HeLa cells transfected as in panel A. Co-precipitated RNA was analyzed by RT-PCR for p53 and small nuclear RNA U6 (control). The asterisk indicates a non-specific co-precipitate. (**C**) Model shows *cis*- and *trans*-regulation of p53 splicing. Exon i9 inclusion is in part determined by 5′ splice site strengths (see Discussion). Moreover, Y14 prevents exon i9 inclusion via association of the EJC with a downstream spliced exon or when it is tethered downstream. After splicing, the EJC may subsequently participate in the translation of p53 mRNA to ensure efficient p53 protein production.

The above data showed that Y14 depletion induced p53β and may additionally increase p53 protein level, although by which mechanism the latter was achieved is unclear. Hence, it was necessary to examine the effect of Y14 overexpression on p53. To our surprise, we observed a minimal increase in the level of p53 protein. The level of p53 mRNA remained unchanged except for the decrease in p53β (Fig. 4A, asterisk, lane 2 compared to lane 1), as observed in Fig. 2B. Similar results were observed in MCF7 cells (Supplementary Fig. 6). This slight p53 increase was also observed upon overexpression of FLAG-eIF4A3, but not of the mRNA cap-binding mutant of Y14 (W73V)^4^ (Fig. 4A, lanes 3, 4). Accordingly, immunoprecipitation and RT-PCR showed that the wild-type Y14, but not the W73V mutant, associated with the p53 mRNA (Fig. 4B). However, we were unable to detect a significant shift of p53 mRNA in polysome fractions upon overexpression of Y14; perhaps this was due to a very minimal effect of Y14 in increasing p53 protein level. Nevertheless, it would be of interest to examine whether cellular signaling may control p53 mRNA translation via Y14 or the EJC. Together with the above results, we assumed that slow or delayed splicing of intron 9 renders exon i9 inclusion, and the EJC, while loaded onto the adjacent ligated exons, can prevent such aberrant splicing and also participates in the subsequent translation of p53 mRNA (Fig. 4C).

### Depletion of Y14 suppresses p21 protein expression

**Figure 5:**
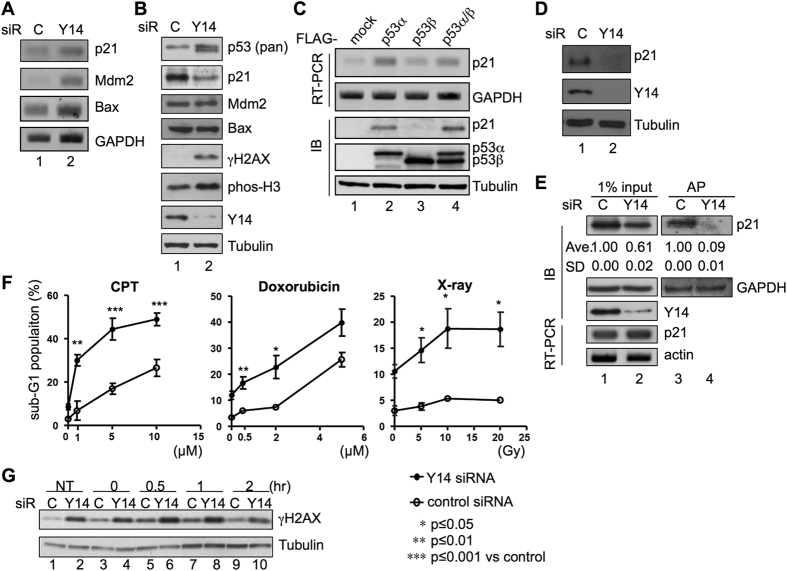
Depletion of Y14 reduces the p21 protein level and increases cell sensitivity to DNA-damaging treatment. (**A**) Immunoblotting of p53 and p21, Mdm2, Bax, phosphorylated H2AX (γH2AX) and H3 (phos-H3), Magoh, Y14, and α-tubulin in control or Y14 siRNA-transfected HeLa cells. (**B**) RT-PCR analysis of p21, Mdm2, Bax, and GAPDH mRNAs in the samples as in panel A. (**C**) The p53α and p53β expression vectors were transfected individually (lanes 1 and 2, respectively) or co-transfected (lane 3) in HeLa cells. RT-PCR shows p21 and GAPDH mRNAs. Immunoblotting (IB) shows p21, transiently expressed p53 proteins, and α-tubulin. **(D)** H1299 cells were transfected with control or Y14 siRNA. Immunoblotting shows p21, Y14 and α-tubulin. (**E**) HeLa cells were transfected with the control or Y14 siRNA followed by metabolic labeling with L-azidohomoalanine. After biotinylation and affinity purification (AP) using streptavidin Sepharose, bound proteins were analyzed by immunoblotting using antibody against p21 and GAPDH. Protein and RNA in 1% lysates were also analyzed by RT-PCR and immunoblotting, respectively. The level of p21 protein in Y14-depleted cells relative to that of control cells was indicated; the averages and standard deviations were obtained from three experiments. (**F**) Line graphs show sensitivity of control (open circle) or Y14-depleted (closed circle) cells to different doses of camptothecin, doxorubicin, or X-rays. The values represent the mean and standard deviation obtained from at least three independent experiments; *p*-values: * < 0.05, ** < 0.01, *** < 0.001. (**G**) Control or Y14-depleted cells were not treated (NT, lanes 1, 2) or irradiated with 2 Gy of X-rays, and cells were collected at the indicated time points post-irradiation (lanes 3–10). Immunoblotting was performed to detect phosphorylated H2AX (γH2AX) and α-tubulin.

The observation that Y14 depletion altered p53 expression (Figs 1, 2, 3) and resulted in G2/M arrest and γH2AX foci accumulation (Supplementary Figs 1 and 7, respectively) prompted us to examine whether Y14 also modulates the expression of p53 targets as well as cellular response to DNA damaging agents. We selected three p53 targets (the cyclin-dependent kinase inhibitor p21^Cip1/Walf1^, E3 ubiquitin-protein ligase Mdm2, and apoptoptic factor Bax) to examine their expression in Y14-depleted HeLa cells. RT-PCR showed that Y14 depletion indeed enhanced their mRNA expression (Fig. 5A), a possible result of p53 activation. Intriguingly, immunoblotting showed that Y14 depletion reduced the level of p21, but did not significantly alter the level of Mdm2 and Bax (Fig. 5B). Nevertheless, Y14 depletion increased the level of γH2AX and phosphorylated histone 3 (Fig. 5B), reflecting G2/M arrest and induction of γH2AX foci.

The above observation prompted us to examine whether p53β had any suppressive effect on p21 protein expression. FLAG-tagged p53α and p53β was individually expressed or co-expressed in HeLa cells. p53β exhibited as a weaker transactivator than p53α with respect to p21 gene expression (Fig. 5C, lanes 2, 3). Co-expression of p53α and p53β, which mimicked Y14 depletion, could still promote the expression of both p21 mRNA and protein (Fig. 5C, lane 4). A similar result was observed using a p53-responsive reporter in TP53-null H1299 cells (Supplementary Fig. 8), indicating that p53β does not compromise the activity of p53α. We also observed that Y14 knockdown reduced p21 protein level in p53-null H1299 cells (Fig. 5D), indicating that Y14 depletion caused downregulation of p21 protein independently of p53.

We have previously found that Y14 depletion selectively reduces protein synthesis^4^. To assess whether Y14 particularly modulates p21 translation, we metabolically labeled newly synthesized proteins in HeLa cells with azidohomoalanine. After the click chemistry reaction, biotinylated proteins were affinity purified for immunoblotting analysis. Figure 5E shows that the level of overall p21 protein was moderately reduced in the Y14-depleted cell lysate (lane 2), whereas newly synthesized p21 was almost not detectable (lane 4). This result suggested that Y14 depletion abolished p21 protein expression likely at the translational level.

### Depletion of Y14 sensitizes cells to DNA damage

p21 primarily functions as a G1/S inhibitor via suppressing the activity of the CDK2 complexes. Nonetheless, p21-deficient cells exhibit delayed G2/M progression and are more vulnerable to DNA damage^22^. p21 also confers anti-apoptotic activity under various cellular conditions^23^ (and references therein). The observation that Y14 depletion reduced p21 protein expression prompted us to examine the genotoxic stress response of Y14-depleted cells. Y14 depletion resulted in a higher basal level of the sub-G1 population (Fig. 5F and Supplementary Fig. 1A), as reported^9^. We treated cells with different doses of CPT or doxorubicin or X-irradiation (IR) and subsequently measured the sub-G1 fraction as an apoptotic index. The result showed that Y14 depletion caused a greater increase in cellular sensitivity to CPT and IR as compared with mock treatment, but it had less impact on the sensitivity to doxorubicin (Fig. 5F). Finally, we examined whether Y14 depletion affects DNA double-strand break repair capacity using immunoblotting of γH2AX. The result showed that the level of γH2AX was constantly higher in Y14-depleted cells than in control both before and after IR treatment (Fig. 5F), suggesting that Y14 may impair DNA repair capacity. Moreover, the possibility that depletion of Y14 or other EJC factors induces co-transcriptional R-loops and subsequent DNA damage^24^ remains to be investigated.

## Discussion

We report for the first time that the EJC factors regulate p53β isoform expression in human cells. p53β is detected in most albeit not all human tissues^13^ but is absent in mouse cells due to the lack of exon i9 (Supplementary Fig. 3). The detailed mechanism for regulation of p53β expression is not fully understood. We postulated that differential strengths of the authentic and cryptic (+329) 5′SSs of intron 9 account for exon i9 selection. The weak authentic 5′ SS site may not compete efficiently with the +329 5′ SS so that exon i9 is activated (Fig. 4C). Moreover, the strong 5′ SS of intron 10 may render its faster splicing than intron 9, leading to deposit of an EJC onto exon 10, which thus prevents exon i9 inclusion or facilitate authentic 5′ SS utilization. Depletion of SRSF3 also activates the expression of p53β^14^. SRSF3 suppresses exon i9 inclusion via binding to its consensus binding elements in exon i9. Caffeine induces p53β by downregulating SRSF3^15^. It would be of interest to know whether the splicing activity of the EJC is modulated by any cellular signaling pathways. Moreover, depletion of the EJC factors impacts different types of alternative splicing^6^,^7^, and thus how the EJC generally selects the targets remains to be investigated.

**Figure 6:**
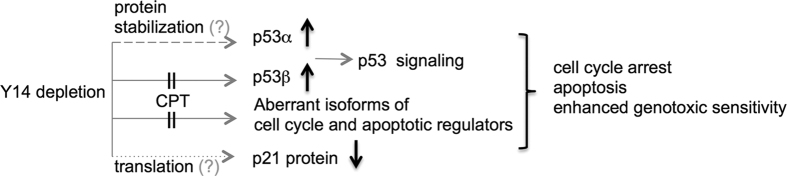
Model depicts the effects of Y14 depletion on the expression of cell cycle and apoptotic factors. Y14 depletion induces the expression of p53β and aberrant isoforms of many other cell cycle and apoptotic regulators via splicing regulation (solid lines). CPT can counteract the effect of Y14 depletion on aberrant splicing. Y14 depletion also increases total p53 protein level possibly by preventing p53 ubiquitination (dashed line), and thus influences p53 signaling. Moreover, Y14 deficiency leads to inefficient p21 protein synthesis (dotted line), leading to reduction of the level of steady p21 protein. The mechanisms of Y14-reguated protein stability control and selective translational control remain further investigation (grey question marks).

Depletion of Y14 increased overall p53 protein levels in various human cells, albeit to different extents, likely by preventing p53 ubiquitination (Fig. 1 and Supplementary Fig. 2). p53 stabilization was not observed in eIF4A3-depleted cells (Fig. 2), suggesting a specific role for Y14 in modulating p53 protein stability, but the underlying mechanism by which Y14 functions in protein stability control remains future investigation. It is notable that SRSF1 stabilizes p53 by abolishing Mdm2-mediated ubiquitination and degradation via its interactions with ribosomal proteins^25^. Thus, Y14 and SRSF1 likely exert opposite roles in modulating p53 stability. Y14 depletion also increased p53 protein level in mouse cells (Supplementary Fig. 3). In fact, Mao *et al*. first reported that Rbm8a haploinsufficiency impairs mouse cortical development and causes microcephaly due to imbalance between proliferation and differentiation of neural progenitors and severe apoptosis of neurons^11^. These phenotypes indeed reflect the effects of Y14 depletion in cultured cells^8^,^9^. Mao *et al*. further reported that haploinsufficiency of individual EJC core factors up-regulated the expression of p53 target transcripts, and that p53 activation accounts for microcephaly possibly by disrupting cell cycle and inducing apoptosis^12^. It is somewhat surprisingly that p53 ablation is sufficient to rescue microcephaly of Rbm8a haploinsufficiency mutants^12^. Y14 depletion indeed activated p53 targets at least at the transcriptional level (Fig. 5A), but it also yielded additional effects, including induction of p53β, aberrant splicing of mitotic and apoptotic factors, and reduction of p21 protein synthesis in human cells (Fig. 6). Depletion of p53 failed to restore p21 protein expression or reduce the sub-G1 population in Y14-depleted cells (Supplementary Fig. 9), reflecting diverse effects of Y14 depletion in human cells, and may also indicate species-specific effect of Y14 depletion al least on alternative splicing of p53.

All together, our results provide biochemical evidence that Y14 impacts p53 signaling in cultured human cells. The details of how Y14 modulates multiple steps of p53 expression and the impact of its absence on cellular functions remain to be investigated.

## Materials and Methods

### Cell culture and transfection

Human cervical cancer HeLa cells, non-small cell lung carcinoma H1299 cells and head and neck squamous carcinoma SAS cells were cultured in Dulbecco’s Modified Eagle’s medium (Invitrogen), breast cancer MCF7 cells in RPMI 1640 medium (Gibco), and colon cancer HCT116 cells in McCoy’s 5A medium (Gibco) at 37 °C and 5% CO_2_. All the mediums contained 10% fetal bovine serum and penicillin/streptomycin/glutamine (Invitrogen). Cell lines were kind gifts of T.-C. Lee, M.-H. Yang, Z.-F. Chang (Taipei). Transfection was performed using Lipofectamine 2000 (Invitrogen). For knockdown experiments, 1 × 10^5^ HeLa cells on 6-well culture plates were transfected with 100 nM siRNA (low GC control, or targeting Y14, Upf1, eIF4AIII or p53). All siRNAs were purchased from ThermoFisher Scientific, and their sequences are listed in Table S1.

### Plasmids

The p53 minigene reporter (p53mini) contained a 4719-bp HeLa cell DNA fragment spanning the 3′ part of intron 6 to the 5′ part of exon 11 (NC_000017.10, CH17: 7584967–7589686) of the human TP5*3* gene. The corresponding DNA fragment was PCR-amplified with the specific primers (Table S2) using HeLa cell genomic DNA as template, and subcloned into the pGEMT vector (Promega). The CMV promoter from pcDNA3.1 (Invitrogen) was inserted into pGEMT-p53mini, resulting the p53mini reporter. The mutant minigenes (p53mini-i9 + 1 m, -i9 + 329 m, -i10weak, -i10dead) were generated by PCR-based site-directed mutagenesis using primers in Table S2. The p53mini-MS2 vector was generated by inserting a 6 × MS2-containing DNA fragment derived from the β6MS2 vector^26^ downstream of exon 10 of a previously reported p53 minigene (a gift of Z.-M. Zheng and C. C. Harris, Bethesda) that spanned exon 7 to 10 (NC_000017.10. CH17: 7584967–7588698)^14^. The expression vectors for FLAG-tagged Y14 and eIF4A3 and MCP-Y14-HA were previously described^3^,^26^. The pcDNA3.1-p53 expression vector was a gift of S.-Y. Shieh (Taipei). To generate the p53β expression vector, we exploited a two-step PCR strategy to replace the C-terminal 62 a.a. coding sequence of p53α with the 10 a.a. p53β-specific sequence. The sequence of all vectors has been confirmed.

### RT-PCR and *in vivo* splicing assay

RNA was isolated by using the TRIzol reagent (Invitrogen) followed by RQ-DNase I (Promega) treatment. RNA (2 μg) was subjected to the reverse transcription (RT) reaction using SuperScript III (Invitrogen) and random hexamer (Invitrogen) or oligo (dT) as primer. Following, 1/40 of the reaction was analyzed by PCR using the specific primers (Table S3). For the splicing reporter assay, 0.5 μg of the p53mini or mutant plasmid was transfected with 100 nM siRNA or 2 μg of FLAG protein expression vector into 1.5 × 10^5^ HeLa cells. For the tethering assay, the p53mini-MS2 vector was co-transfected with the MCP-Y14-HA expression vector. For RT-PCR, primers CMV and P2 were used. For Southern blotting, the RT-PCR products were separated on 6% denaturing polyacrylamide gel and detected by ^32^P-labeled P2 probe (Table S3). RNP immunoprecipitation was performed as previously described^4^.

### Immunoblotting

Immunoblotting analysis was essentially as described in Chuang *et al*. (2013). Antibodies included monoclonal anti-α-tubulin (Neo Marker), Y14 (abcam) and p53 (DO-1; Santa Cruz), and polyclonal anti-p53 (pan from Proteintech, and C-19 from Santa Cruz), Y14 (Bethyl), eIF4AIII (Proteintech), Magoh (Abcam), Upf1 (Cell Signaling), p21 (Santa Cruz), γH2AX (Millipore), phospho-histone 3 (Cell signaling), HA (Bioman) and FLAG epitope (Sigma).

### Metabolic labeling and affinity purification of newly synthesized proteins

HeLa cells were transfected with the control or Y14 siRNA and incubated 48 hrs post-transfection. Metabolic labeling of polypeptides with L-azidohomoalanine was subsequently performed using the Click-iT Protein Reaction Buffer kit (Invitrogen) as described^4^. The reactions were incubated with streptavidin Sepharose (GE Healthcare Life Science) overnight at 4 °C followed by extensive wash with 0.5% SDS-containing phosphate-buffered saline (PBS). Purified proteins were analyzed by immunoblotting.

### Immunoprecipitation-RT-PCR

HeLa cells were transfected with a FLAG-tagged protein expression vector for 48 hrs. Cell lysates were prepared and incubated with anti-FLAG M2 beads (Sigma) in NET-2 buffer containing 50 mM Tris-HCl (pH 7.4), 150 mM NaCl, and 0.05% NP-40 at 4 °C for 2 hrs. After washing with NET-2 buffer, RNA was recovered using TRIzol reagent, and subjected to RT-PCR analysis using the primers shown in Table S3.

### DNA damage treatment and sub-G1 analysis

HeLa cells were transfected with control or Y14 siRNA as above. Thirty-two hrs post-transfection, cells were treated with CPT or doxorubicin for 16 h or irradiated with X-rays followed by incubation for 16 h before harvest. Subsequently, cells were trypsinized, washed with PBS and fixed in 70% ethanol overnight at −20 °C. After fixation, cells were subjected to staining in a solution containing 1 mg/ml propidium iodide, 0.1% triton X-100 and 10 mg/ml RNase A in PBS. Samples were analyzed by using FACSCanto-6color (BD Biosciences).

## Additional Information

**How to cite this article:** Lu, C.-C. *et al*. Y14 governs p53 expression and modulates DNA damage sensitivity. *Sci. Rep.* **7**, 45558; doi: 10.1038/srep45558 (2017).

**Publisher's note:** Springer Nature remains neutral with regard to jurisdictional claims in published maps and institutional affiliations.

## Supplementary Material

Supplementary Information
