# Supplementary material for: Y14 governs p53 expression and modulates DNA damage sensitivity
Source: Sci Rep. 2017 Mar 31;7:45558. doi: 10.1038/srep45558 (PMC5374521; doi:10.1038/srep45558)
Supplement: Supplementary Information [file srep45558-s1.pdf]

Y14 governs p53 expression and modulates DNA damage sensitivity

Chia-Chen Lu[1,2], Chi-Chieh Lee[2], Ching-Tzu Tseng[2], Woan-Yuh Tarn[1,2*]
[1]Taiwan International Graduate Program in Molecular Medicine, National Yang-Ming University and Academia Sinica, [2]Institute of Biomedical Sciences, Taipei, Taiwan

Corresponding author:
Woan-Yuh Tarn
Institute of Biomedical Sciences
Academia Sinica
128 Academia Road, Section 2, Nankang
Taipei 11529, Taiwan
E-mail: wtarn@ibms.sinica.edu.tw

Supplemental Materials and Methods

Cell culture

HeLa and H1299 cells were cultured as described in the main text. Mouse neuroblastomaneuro2a (N2A) cells were cultured under the same condition as HeLa cells (see the main text).

Plasmids

The expression vector of FLAG-Upf1-DN has been described in Lee et al. (2010) [1].The pGL3-3PBS-Luc (hereafter p53-Luc) plasmid containing three repeats of the p53-binding sequence (AGGCATGTCT) was obtained from Y.-S. Lin (Taipei) (Hsu et al. 1995) [2]. The expression vectors respectively encoding HA-Ub and HA-Ub GGAA were obtained from Dr. Y.-L. Lin (Taipei).

Luciferase reporter assay

The p53-Luc (2 μg) and control pRL-SV40 (Promega; 0.2 μg) expressing Renilla luciferase were co-transfected with p53α (1 μg) and p53β (0, 0.25, 0.5 or 1 μg) into H1299 cells (1.5×10^5 cells). Cells were harvested 24 hrs post-transfection. The cell lysates were subjected to the luciferase assay using the Dual-Luciferase Reporter Assay system (Promega). Firefly luciferase activity was normalized to Renilla luciferase activity for each sample.

Cell cycle analysis and apoptosis assay

HeLa cells were transfected with Y14 siRNA for 48 hour. Cells were then trypsinized, washed in cold PBS and fixed in 70% ethanol overnight in -20˚C. After fixation, cells were washed in PBS and resuspended in PBS containing 1 mg/ml propidium iodide, 0.1% triton X-100 and 10 mg/ml RNAse A for staining. After 30 min in dark at room temperature, cells were analyzed by using FACSCanto-6color (BD, Biosciences). Y14 knockdown cells were then trypsinized, washed in cold PBS and resuspended in staining buffer containing Annexin V and propidium iodide (BD, Biosciences). After 15 minute of incubation in dark at room temperature, samples were analyzed by flow cytometry.

Immunofluorescence

Cells were grown on slides, fixed with 3% paraformaldehyde for 30 min, and permeabilized with 0.5% Triton X-100 for 5 min. After fixation, slides were blocked in 3% BSA in PBS for 1 h at room temperature, and incubated with the following primary antibodies: polyclonal anti-γH2AX (Ser-139; Millipore) or monoclonal anti-Y14

(Abcam) for 1h. Secondary antibodies conjugated with Alexa 488 or Rhodamine (MP Biomedicals) were added for 1 h. Slides were then stained with DAPI for 5 minutes and mounted. Images were acquired with confocal (Zeiss LSM700).

Supplemental References

1 Lee, K. M., Hsu, I. W. & Tarn, W. Y. TRAP150 activates pre-mRNA splicing and promotes nuclear mRNA degradation. *Nucleic Acids Res* **38**, 3340-3350 (2010).

2 Hsu, Y. S. *et al.* Transcriptional regulation by p53 - functional interactions among multiple regulatory domains. *J Biol Chem* **270**, 6966-6974 (1995).

Supplemental Tables

Supplemental Table S1: siRNAs

Gene	5' to 3'
Y14	GGGUAUACUCUAGUUGAAUAUGAAA
Upf1	CCCAACCCGAUAAACCGAUGUUCU
Magoh	ACAAGGGCAAGUUCGGCCACGAGUU
eIF4A3	UUGAGUUUCACGAACCUGAAUAUCC
p53	GAAAUUUGCGUGUGGAGUA

Supplemental Table S2: Primers used for plasmid construction

Primer	Dir.	5' to 3'
p53intron6-HindIII	F	AAGCTTGGGCCCACCTCTTACCGAT
p53exon11-KpnI	R	AGCGGTACCAGGCCCTTCTGTCTTGAAC
i9+1m	F	CTGGATGGAGAATATTTCACCCTTCAGGTAAGTAGTCTTGGGACCTCTTATC
	R	GATAAGAGGTCCCAAGACTACTTACCTGAAGGGTGAAATATTCTCCATCCAG
i9+329m	F	GTAAGTTGAAAATATTGTCCTATGAAAATGGATTTAATAC
	R	GTATTAAATCCATTTTCATAGGACAATATTTTCAACTTAC
i10weak	F	CCAGGGGGGAGCAGGGCTCACTCCAGGTCCTAGACCTCAGCCCCTTCCTGGCCC
	R	GGGCCAGGAAGGGGCTGAGGTCTAGGACCTGGAGTGAGCCCTGCTCCCCCCTGG
i10dead	F	CCAGGGGGGAGCAGGGCTCACTCCAGATAAATGACCTCAGCCCCTTCCTGGCCC
	R	GGGCCAGGAAGGGGCTGAGGTCATTTATCTGGAGTGAGCCCTGCTCCCCCCTGG
E10-XhoI-MS2	F	ACG CTCGAG TAGAAAACATGAGGATCACCC
MS2-XhoI	R	GCGCCTCGAGAATAGGGCCCTCTAG
p53β-P1	F	ATTGTTAACTCGAGGACTACAAGGACGA
	R	GGTCTGGTCCTGAAGGGTGAAATATTCTC
p53β-P2	F	AAAAGAAAATTGTTAACTCGAGGACTAC
	R	TGAAAGCTGGTCTGGTCCTGAAGGGTGA

Supplemental Table S3: RT-PCR primers

Gene or site	Dir.	5' to 3'
p53β (P3)	F	CTTTGAGGTGCGTGTTTGTGC
(P4)	R	TTGAAAGCTGGTCTGGTCCTGA
p53 total (P1)	F	CTCACCATCATCACACTGGAA
(P2)	R	TCATTCAGCTCTCGGAACATC
Y14	F	GATTATGACAGCGTGGAGCA
	R	GTCTCCGGTCTGGACTTCTG
Bcl-x	F	ATGGCAGCAGTAAAGCAAGCG
	R	TCATTTCCGACTGAAGAGTGA
p21	F	GGAGACTCTCAGGGTCGAAA
	R	GGATTAGGGCTTCCTCTTGG
SF1	F	ATTCCCCCTGGACTTACTCG
	R	ACTCACACGTGTTGCTGGAG
Bax	F	GGACGAACTGGACAGTAACATGG
	R	GCAAAGTAGAAAAGGGCGACAAC
Mdm2	F	GAGGGCTTTGATGTTCCTGA
	R	GCTACTAGAAGTTGATGGC
PNN	F	AGAAGAGAATCACGCCAGGA
	R	CCGCTTTTGCCTTTCAGTAG
EIF2S2	F	CCCAGCCTTCAGAAACAAAA
	R	TTCTCCCCAGCAACCATATC
CMV	F	CCGCGG GATTAAGCT TCTTGG
p53β specific (P5)	R	CTTTTTGAAAGCTGGTCTGGTC
p53γ specific (P6)	R	CCATCGTAAGTCAAGTAGC
GAPDH	F	CGGAGTCAACGGATTTGGTCGTATG
	R	AGCCTTCTCCATGGTGGTGAAGAC
U6	F	CTCGCTTCGGCAGCACA
	R	AACGCTTCACGAATTTGCGT
p53(mouse)	F	CTCACCATCATCACACTGGAA
	R	TCATTCAGCTCCCGGAACATC
GAPDH (mouse)	F	GTCGTGGAGTCTACTGGTGT
	R	TACTTGGCAGGTTTCTCCAG
p53 exon 9	R	GGAGCTGGTGTTGTTGGGCAG

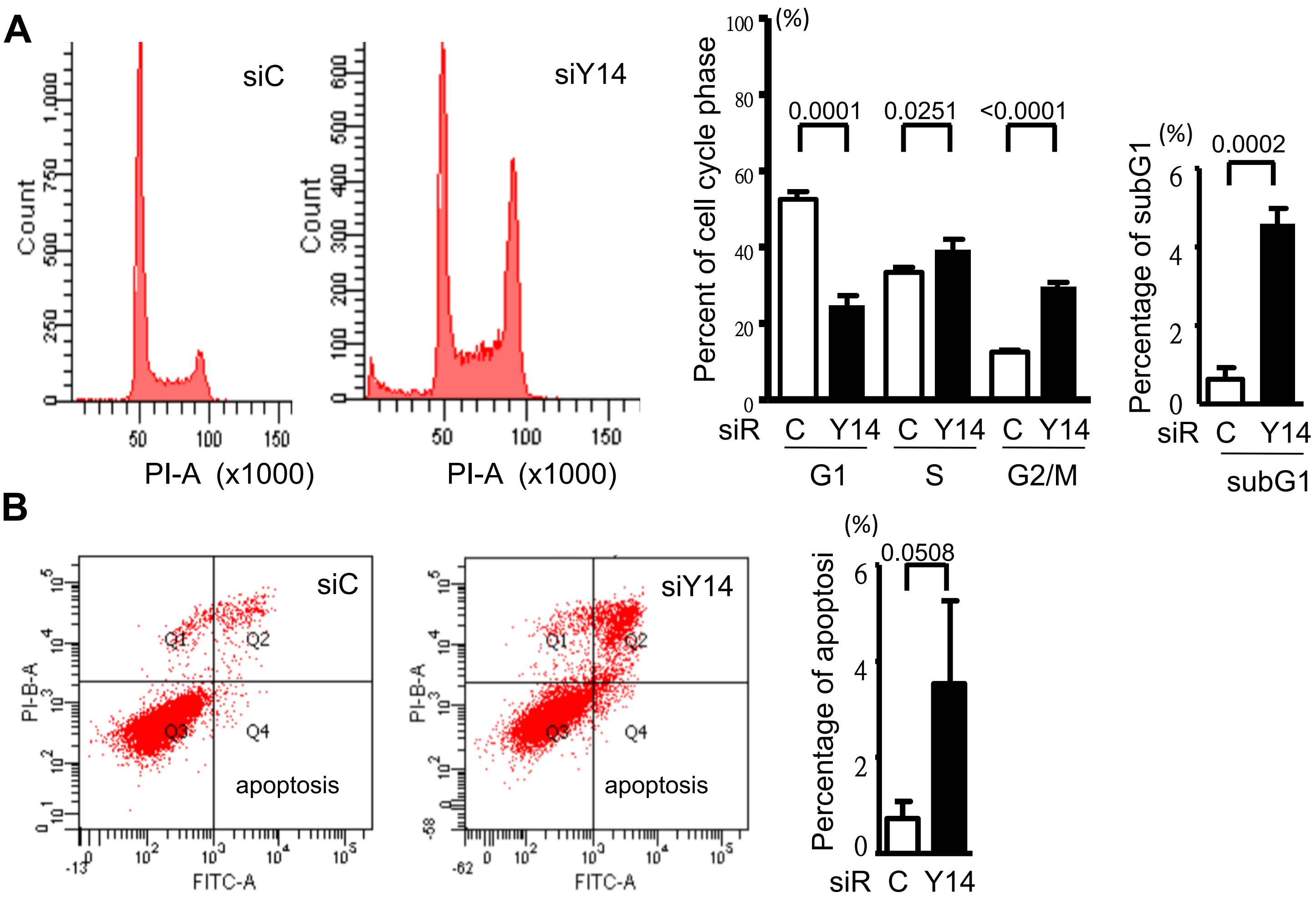

Figure S1. Y14 knockdown induces cell cycle arrest and apoptosis.
(A) HeLa cells were transfected with control or Y14 siRNA. Cells were stained with propidium iodide (PI) and the DNA content was determined by flow cytometry. A representative result is shown at left. Bar graphs show percentage of control or Y14-depleted cells at each cell cycle phase and sub-G1 population. (B) Control or Y14-knockdown cells were stained with PI and Annexin-V, followed by flow cytometry analysis. Left panels show representative flow cytometric analysis result. Bar graph shows percentage of apoptotic (PI-negative/Annexin V-positive; Q4) cells. Mean and standard deviation were obtained from three independent experiments; *p* vales are indicated above.

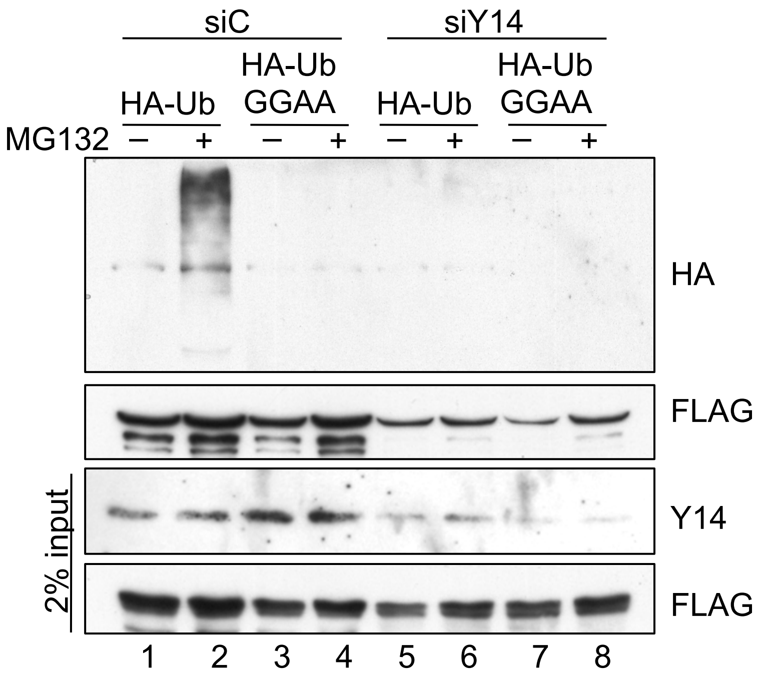

Figure S2. Y14 knockdown prevents ubiquitination of p53.
HeLa cells were co-transfected with siRNA (siC or siY14) and the expression vector of FLAG-tagged p53 and HA-tagged ubiquitin (HA-Ub; wild-type or mutant GGAA). Cells were mock-treated or treated cells with the proteasome inhibitor MG132 for 16 hrs. FLAG-p53 was immunoprecipitated by using M2 (anti-FLAG) agarose. Input (2%) and immunoprecipitates were analyzed by immunoblotting using antibodies as indicated.

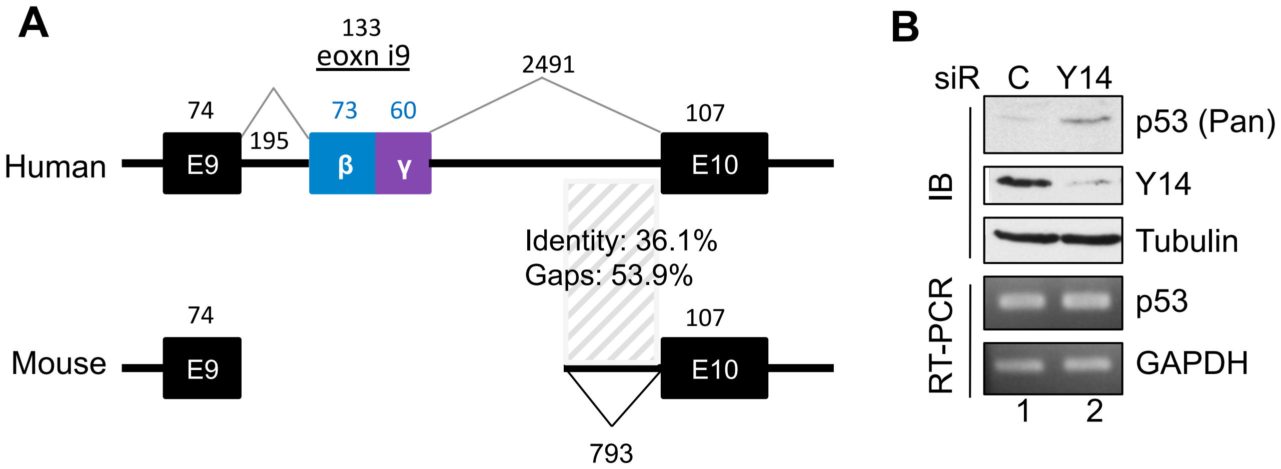

Figure S3. Y14 knockdown increases p53 protein level in mouse N2A cells.
(A) Schematic diagram shows intron 9 of human and mouse TP53 genes. The human p53 intron 9 is 2819-bp, containing a 133-bp exon i9 in its 5' part. The mouse p53 intron 9 (793 bp) exhibits ~36% sequence identity with the 3' part of its human counterpart. (B) N2A cells were transfected with control or Y14 siRNA. Immunoblotting of p53, Y14 and α-tubulin, and RT-PCR of p53 and GAPDH were performed.

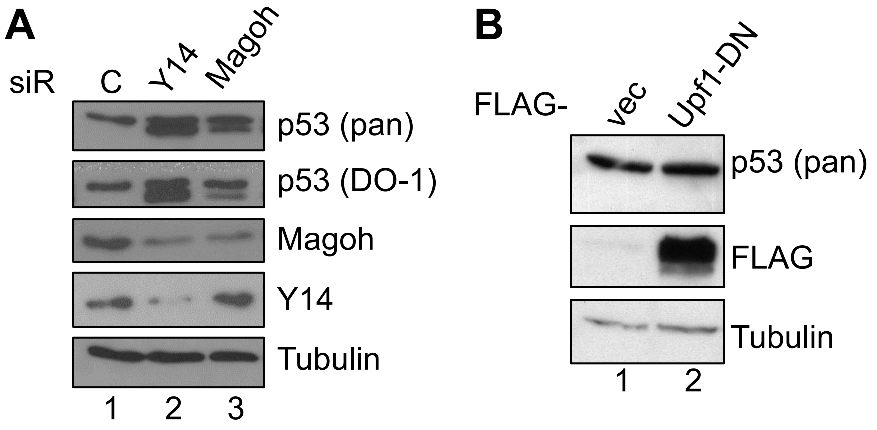

Figure S4. Effect of the EJC and NMD factors on p53β expression.
(A) HeLa cells were transfected with control or Y14 or Magoh siRNA. (B) HeLa cells were transfected with the empty or dominant negative Upf1 (Upf1-DN) expression vector. For both panels, cell lysates were subjected to immunoblotting analysis using antibodies as indicated.

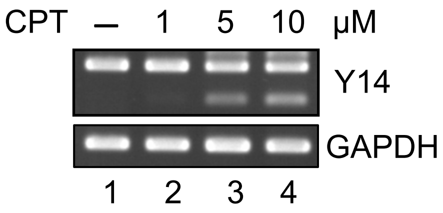

Figure S5. Effects of RNA pol II and topoisomerase inhibitors on splicing.
HeLa cells were treated with different doses of CPT for 16 hrs. RT-PCR shows exon 3 skipping of Y14.

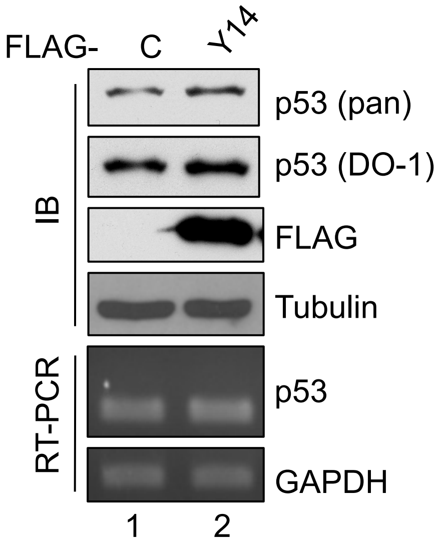

Figure S6. Overexpression of Y14 slightly increases the level of p53 protein in MCF7 cells.
MCF cells were transfected with the empty or FLAG-Y14 expression vector. Immunobotting shows p53, FLAG-Y14 and α-tubulin. RT-PCR shows p53 and GAPDH.

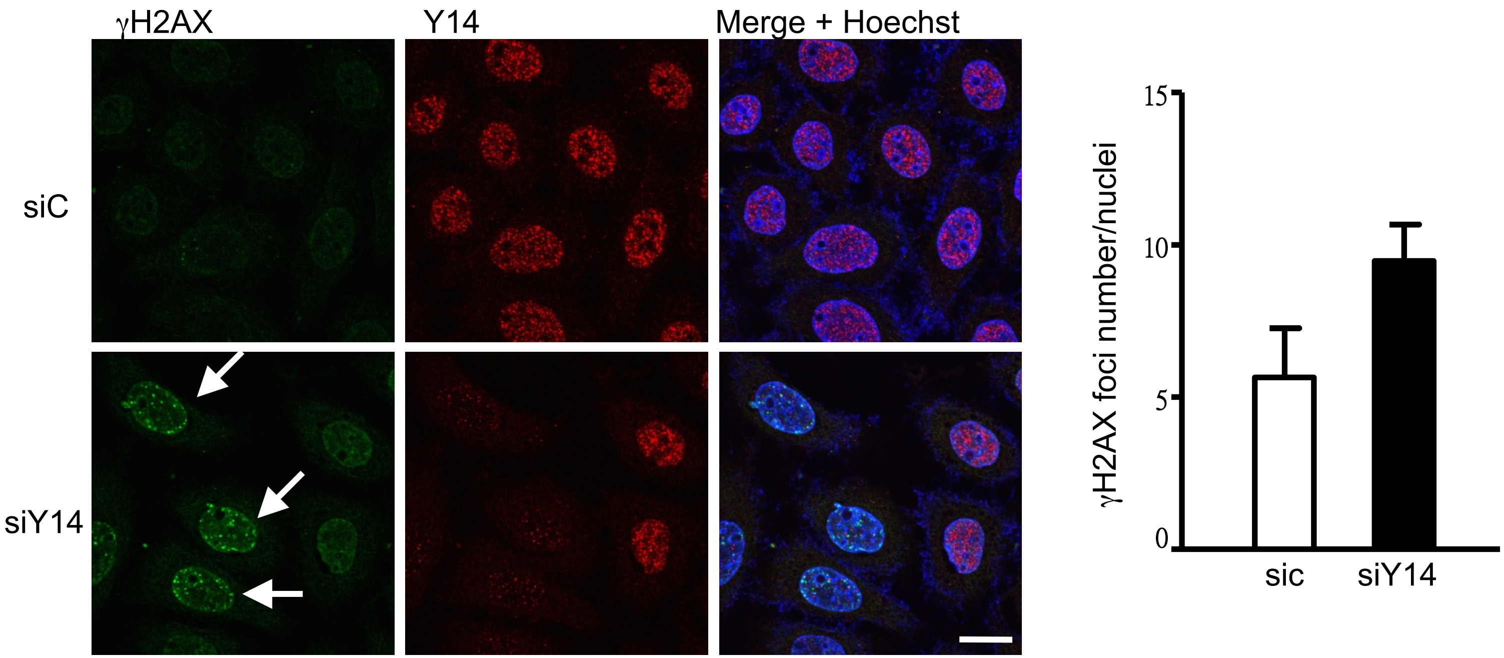

Figure S7. Y14 knockdown causes the accumulation of DNA damage foci.
Representative images show anti-γH2AX immunofluorescence of siRNA (control or Y14)-transfected HeLa cells. Bar graph shows the number of γH2AX foci in control and Y14-depleted cells. Average was obtained from three independent experiments; ~300 cells were counted in each experiment.

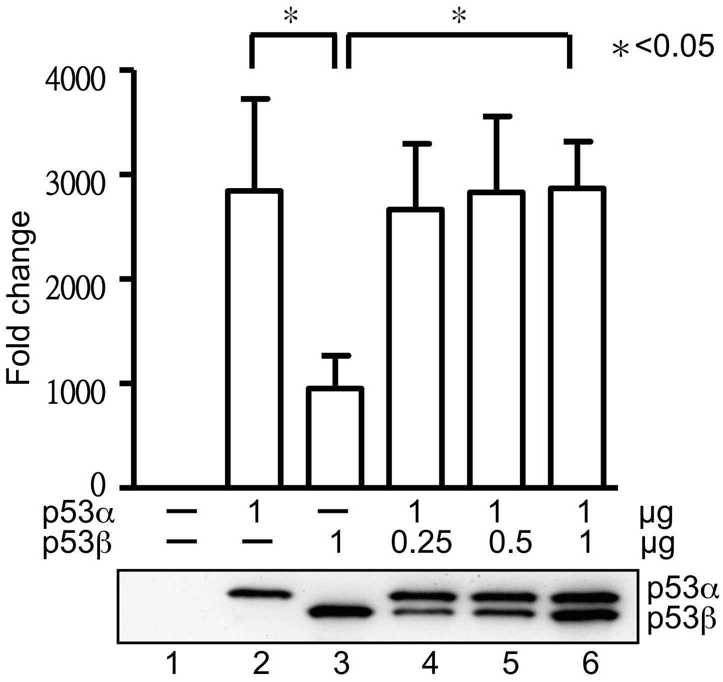

Figure S8. p53β does not interfere with the transactivation activity of p53α.
H1299 cells were transiently co-transfected with the luciferase reporters (p53-Luc and pRL-SV40) with the p53α or β expression vector; vector amounts were as indicated. Bar graph shows activation folds; average and standard deviation were from 3 experiments. Immunoblotting shows transiently expressed p53 protein isoforms.

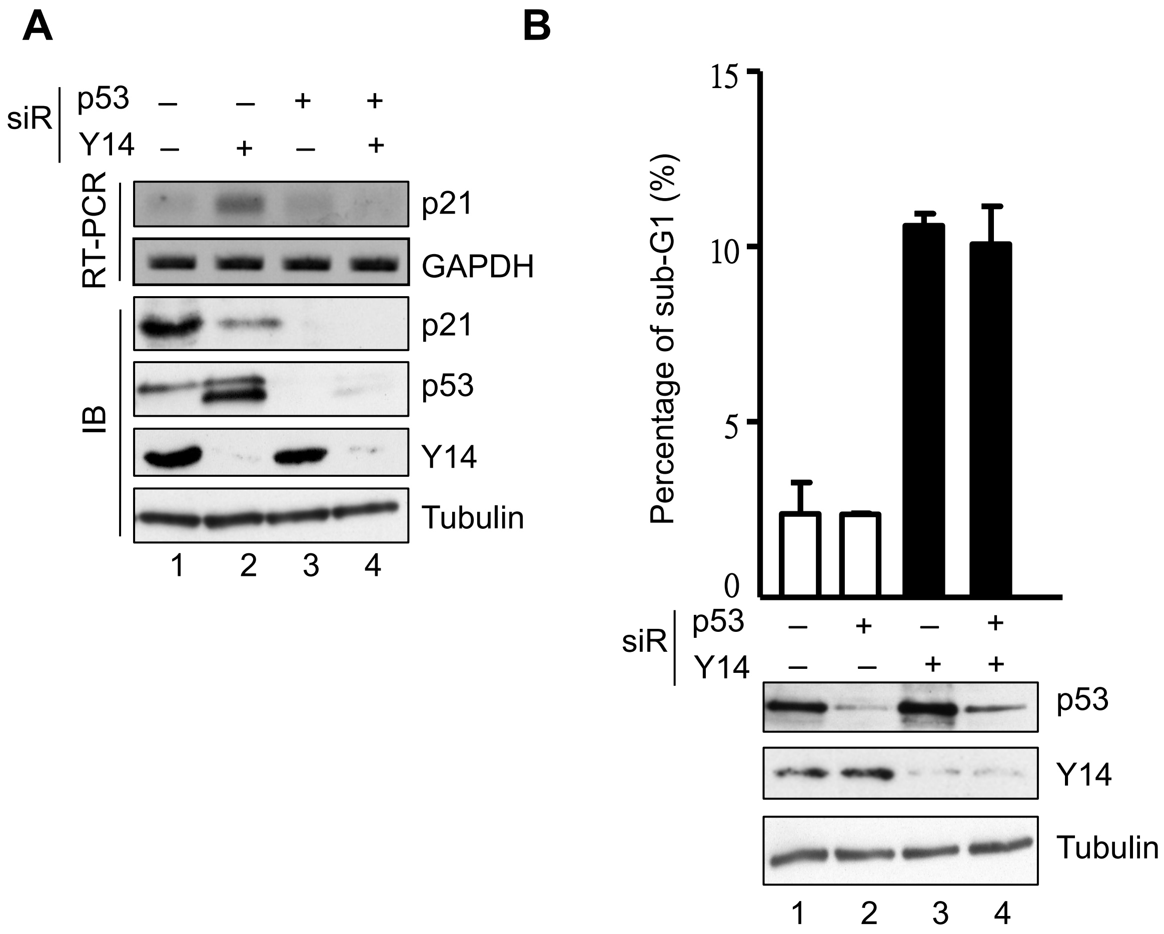

Figure S9. Knockdown of p53 did not reduce sub-G1 population of Y14 depleted cells.
(A) HeLa cells were transfected with control or Y14 siRNA together with or without p53 siRNA. RT-PCR shows p21 and GAPDH. Immunoblotting shows p21, p53, Y14 and α-tubulin. (B) HeLa cells were transfected as in panel A, and then subjected to the sub-G1 analysis as in Fig. S1. Immunoblotting shows Y14, p53 and α-tubulin.
